# Allergic contact dermatitis to benzoxonium chloride contained in an antiseptic solution

**DOI:** 10.1111/cod.14160

**Published:** 2022-05-23

**Authors:** Aurélie Hsieh, Olivier Sorg, Pierre‐André Piletta

**Affiliations:** ^1^ Division of Dermatology and Venereology Geneva University Hospital Geneva; ^2^ Clinical Pharmacology and Toxicology Unit University of Geneva Geneva Switzerland

**Keywords:** allergic contact dermatitis, antiseptic, benzoxonium, benzoxonium chloride, case report, disinfectant

Contact dermatitis to antiseptics is well known and mainly imputed to chlorhexidine, povidone iodine and benzalkonium chloride. Benzoxonium chloride (BXC; CAS no. 19379‐90‐0) is quaternary ammonium with antibacterial, antiviral and antimycotic properties sometimes found in antiseptics. We report here two cases of allergic contact dermatitis caused by BXC.

## CASE 1

For many years, a 6‐year‐old male child had experienced oedematous dermatitis with wound healing impairments after treatment of minor injuries with antiseptics. A “paediatric” baseline series adapted from the American Contact Dermatitis Society^1^ containing chlorhexidine digluconate 0.5% aq. and benzalkonium chloride 0.1% aq. (all from Chemotechnique Diagnostics) and several of the child's own products were patch tested. The patch tests showed after an occlusion time of 48 h a doubtful reaction to chlorhexidine digluconate 0.5% aq. (D2 ‐; D4 ?+) and a positive reaction to Merfen aqueous solution (Verfora), tested “as is” (D2 ++; D4 ++).

**FIGURE 1:**
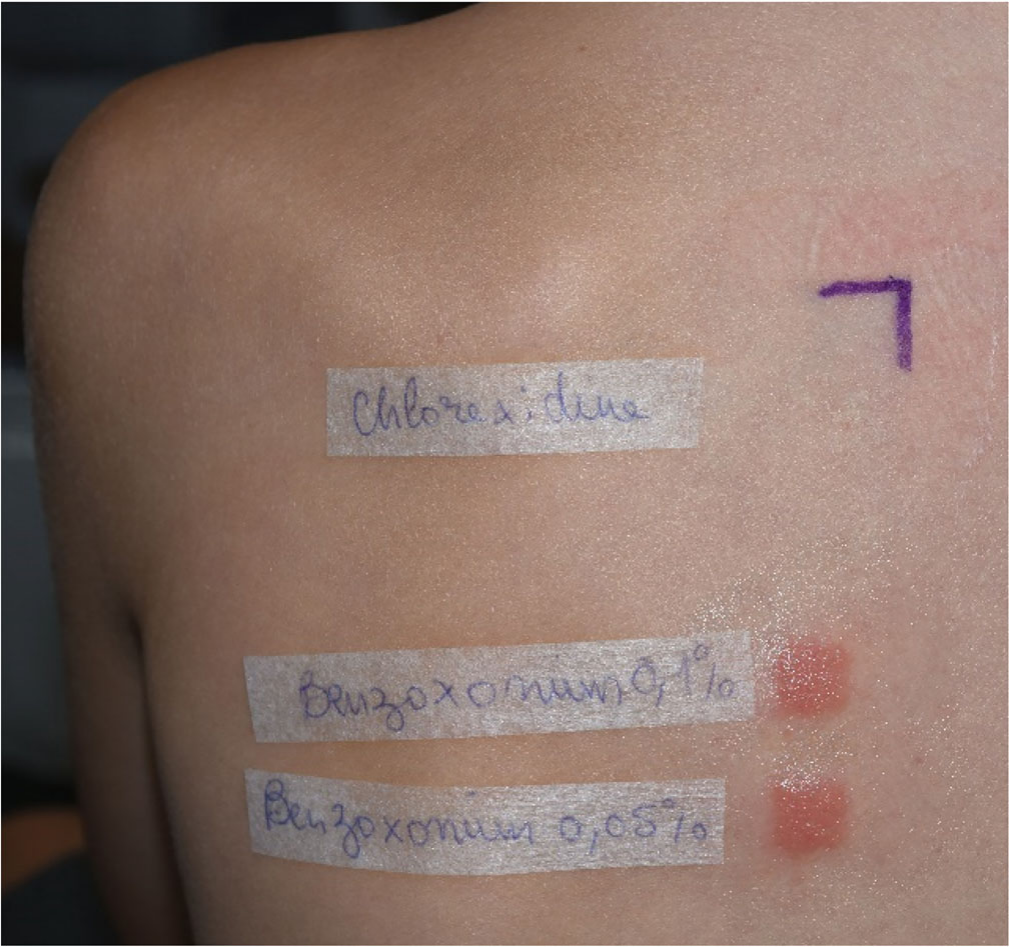
Case 1: positive reaction (+++) to benzoxonium chloride 0.1% aq. and 0.05% aq. at D4

Additional patch testing with BXC contained in Merfen aqueous solution (Verfora) and kindly provided by Verfora resulted in positive reaction to BXC 0.1% aq. (D2 ++; D4 +++) and BXC 0.05% aq. (D2 ++; D4 +++). A repeated patch testing with chlorhexidine digluconate 0.5% aq. remained negative (Figure 1). The diagnosis of allergic contact dermatitis to BXC was established. After avoidance of the allergen, wound healing delay and dermatitis did not recur.

## CASE 2

**FIGURE 2:**
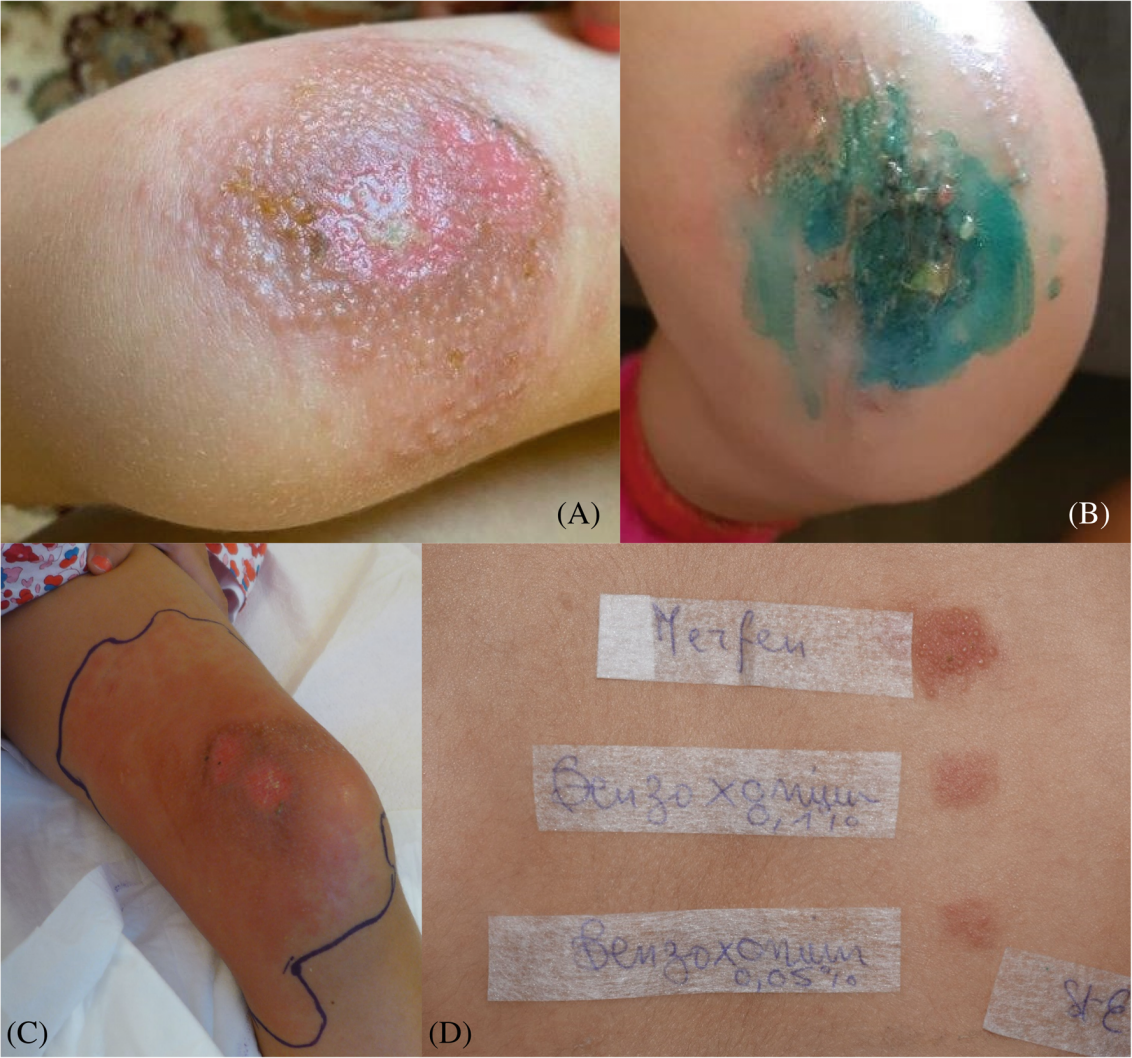
Case 2. (A,B) Vesiculobullous reaction of the knees after using antiseptics (C) Persistent dermatitis. (D) Positive patch test reactions to Merfen aqueous solution (Verfora) and benzoxonium chloride 0.1% and 0.05% aq.

A 4‐year‐old female child presented four episodes of localized vesiculobullous rash after minor trauma to the knee treated with different types of antiseptics. Many antiseptics and topical antibiotics had been applied without resolving the rash. She was even hospitalized for a course of IV antibiotics because of the suspicion of an infection. Improvement was only observed with corticosteroid application. Patch tests with a “paediatric” baseline series adapted from the American Contact Dermatitis Society^1^ and some of the child's own products showed after an occlusion time of 48 h, a strong reaction to Merfen aqueous solution (Verfora), tested “as is” (D2 +++; D4 +++) and BXC 0.1% aq. and 0.05% aq. (D2 +; D4 ++) tested at the same time. Chlorhexidine digluconate 0.5% aq. and benzalkonium chloride 0.1% aq. were negative (Figure 2). No episodes of vesiculobullous lesions recurred after eviction of Merfen aqueous solution (Verfora) and BXC.

## DISCUSSION

BXC is used for its antiseptic properties and is a constituent of Merfen aqueous solution (Verfora), 1 ml contain 5 mg of chlorhexidine digluconate and 1 mg of BXC. It is available over‐the‐counter and widely employed in Switzerland.

Only three other cases of allergic contact dermatitis to BXC have been reported (Table 1). We describe here two new cases of allergic contact dermatitis to BXC in children. The two patients had no concomitant reaction to benzalkonium chloride, another quaternary ammonium. Domiphen bromide was not tested.

**TABLE 1:** Published cases of allergic contact dermatitis to benzoxonium chloride

Case	Product applied containing BXC	Age/sex	Clinical features	Patch tests results	Comments
De Groot et al.^2^	Ointment intended for veterinary use, used to treat rhagades of the fingers by the patient	58/F	Erythematous rash of the face and neck without affecting her hands	Ointment (D2 ++, D3 ++) BXC 0.1% aq. (D2 +, D3 ++, D7 ++) and BXC 0.01% aq. (D2? +, D3 + and D7 +) Domiphen bromide 0.1% aq. (D2 +, D3 +, D7 ?+) Benzalkonium chloride 0.1% aq. (D2 +, D3 +, D7 ?+)	30 Controls tested with BXC 0.1% aq., 2 showed a doubtful reaction (?+) at D2 and D3, no reactions to BXC 0.01% aq. Patch tests positive for two others quaternary ammonium compounds (domiphen bromide and benzalkonium chloride)
Bruynzeel et al.^3^	Emollient for dry skin intended for veterinary use	43/M	Face, arms, hands, left shoulder and axilla dermatitis	Emollient (D2 +++, D3 +++) BXC 0.02% aq. (D2 ++, D3 ++) Wool alcohols (D2 +++, D3 +++)	20 Controls tested with BXC 0.02% aq. were negative The emollient did not contain lanolin or wool alcohols
Diaz‐Ramon L et al.^4^	Spanish topical corticosteroid containing hydrocortisone aceponate and BXC used for seborrheic dermatitis	37/F	Scalp, forehead and retroauricular dermatitis	Topical corticosteroid (D4 +++) BXC 0.1% aq. (D2 ++, D4 ++)	Other components of this cream tested negative 20 Controls tested with 0.1% BXC aq. without reaction

Abbreviation: BXC, benzoxonium chloride.

BXC is a highly electrophilic hapten and can stabilize nuclear factor erythroid 2‐related factor 2, a transcription factor that plays a protective role in contact hypersensitivity. It works as a key regulator of inducible cellular defence and down‐regulates cellular oxidative stress and inflammatory pathways within various cell types, including innate immune cells.^5^ In case of allergic contact dermatitis, however, this protective mechanism seems not sufficient to counteract the immune response induced by BXC.

In conclusion, wound healing delay or worsening of wounds with accompanying dermatitis might be caused by allergic contact dermatitis to disinfectants. The diagnosis can be easily missed and may lead to extensive investigations or empirical treatment as in our second case. Allergic contact dermatitis to BXC should be suspected and this allergen eventually added to the disinfectant series.

## AUTHOR CONTRIBUTIONS

**Aurélie Hsieh:** Methodology; writing – review and editing; conceptualization; investigation; writing – original draft. **Olivier Sorg:** Writing – review and editing. **Pierre‐André Piletta:** Conceptualization; investigation; methodology; validation; visualization; supervision.

## CONFLICT OF INTEREST

The authors declare no conflict of interest.
